# TRAP1 modulates mitochondrial biogenesis via PGC-1α/TFAM signalling pathway in colorectal cancer cells

**DOI:** 10.1007/s00109-024-02479-9

**Published:** 2024-08-29

**Authors:** Giuseppina Bruno, Michele Pietrafesa, Fabiana Crispo, Annamaria Piscazzi, Francesca Maddalena, Guido Giordano, Vincenza Conteduca, Marianna Garofoli, Almudena Porras, Franca Esposito, Matteo Landriscina

**Affiliations:** 1 Medical Oncology and Biomolecular Therapy Unit, Department of Medical and Surgical Sciences, University of Foggia, Viale Pinto 1, 71122 Foggia, Italy; 2 Laboratory of Pre-Clinical and Translational Research, IRCCS, Referral Cancer Center of Basilicata, 85028 Rionero in Vulture, Potenza Italy; 3 Department of Biochemistry and Molecular Biology, Faculty of Pharmacy, Complutense University of Madrid, 28040 Madrid, Spain; 4 Health Research Institute of the Hospital Clínico San Carlos (IdISSC), 28040 Madrid, Spain; 5 Department of Molecular Medicine and Medical Biotechnology, University of Naples Federico II, 80131 Naples, Italy

**Keywords:** TNF receptor-associated protein 1, Mitochondrial biogenesis, Colorectal cancer, Metabolism, Peroxisome proliferation-activated receptor gamma coactivator α1-alpha, Transcription factor A mitochondrial

## Abstract

**Abstract:**

Metabolic rewiring promotes cancer cell adaptation to a hostile microenvironment, representing a hallmark of cancer. This process involves mitochondrial function and is mechanistically linked to the balance between mitochondrial biogenesis (MB) and mitophagy. The molecular chaperone TRAP1 is overexpressed in 60–70% of human colorectal cancers (CRC) and its over-expression correlates with poor clinical outcome, being associated with many cancer cell functions (i.e. adaptation to stress, protection from apoptosis and drug resistance, protein synthesis quality control, metabolic rewiring from glycolysis to mitochondrial respiration and vice versa). Here, the potential new role of TRAP1 in regulating mitochondrial dynamics was investigated in CRC cell lines and human CRCs. Our results revealed an inverse correlation between TRAP1 and mitochondrial-encoded respiratory chain proteins both at transcriptional and translational levels. Furthermore, TRAP1 silencing is associated with increased mitochondrial mass and mitochondrial DNA copy number (mtDNA-CN) as well as enhanced MB through PGC-1α/TFAM signalling pathway, promoting the formation of new functioning mitochondria and, likely, underlying the metabolic shift towards oxidative phosphorylation. These results suggest an involvement of TRAP1 in regulating MB process in human CRC cells.

**Key messages:**

- TRAP1 inversely correlates with protein-coding mitochondrial gene expression in CRC cells and tumours.
- TRAP1 silencing correlates with increased mitochondrial mass and mtDNA copy number in CRC cells.
- TRAP1 silencing favours mitochondrial biogenesis in CRC cells.

**Supplementary Information:**

The online version contains supplementary material available at 10.1007/s00109-024-02479-9.

## Introduction

Despite important advancements in cancer therapy, colorectal cancer (CRC) still represents the second leading cause of cancer death worldwide [1, 2], suggesting that new targetable biomarkers need to be identified to overcome CRC onset and progression. Supporting cellular adaptation to harsh microenvironment, metabolic rewiring is considered a hallmark of cancer and a novel therapeutic target in many solid tumours [3–5]. Indeed, cancer cells remodel their metabolism to support the high demand on nutrients for building blocks and energy production [6] and often present an increased rate of glycolysis and lactate production even in the presence of oxygen, a phenomenon known as Warburg effect [7]. The Warburg effect has been considered for many years a direct consequence of damage mitochondria; however, it was demonstrated that cancer cell mitochondria maintain their functionality, which confers high levels of cellular plasticity and fast adaptation to hostile environmental conditions, including hypoxia and nutrient depletion [8, 9]. Mitochondria play a central role in many biological functions (i.e. bioenergetics, biosynthesis, calcium and redox homeostasis, apoptosis) [10] as well as in all steps of tumorigenesis [11, 12], with both mitochondrial biogenesis (MB) and mitophagy involved in many oncogenic signalling pathways [12].

Mitochondrial function depends on molecular chaperones for both respiratory complexes formation and clearance of misfolded proteins [13]. In this context, TNF receptor-associated protein 1 (TRAP1) is a member of the HSP90 chaperone family, with a prevalent mitochondrial localization, overexpressed in several human malignancies and, specifically, in ~ 60% of human CRCs. Its expression is directly associated with poor clinical outcome, protection from apoptosis, drug resistance and mitochondrial respiration inhibition [14–16]. TRAP1 is also involved in cell adaptation to stress environmental conditions favouring glycolytic metabolism rather than oxidative phosphorylation (OXPHOS) in both CRC cell lines and CRC patient-derived spheroids [17]. Furthermore, it was suggested that TRAP1 expression levels influence mitochondrial architecture of human neuroblastoma cells and tumour metastasis in vivo [18].

Based on this rationale, the present study addressed the hypothesis that TRAP1 may be involved in the regulator of mitochondrial dynamics in human CRC cell lines.

## Materials and methods

### Cell cultures, reagents and cell transfection procedures

HCT116 (RRID:CVCL_0291; ATCC number CCL-247) and SW48 (RRDI:CVCL_1724; ATCC number CCL-231) cell lines were purchased from the American Type Culture Collection (ATCC). HCT116 cell line was cultured in McCoy’s 5A medium (cat. n^o^ 26600023, Gibco; Thermo Fisher Scientific, Inc.) with 10% fetal bovine serum (cat. n^o^ 10270106, Gibco; Thermo Fisher Scientific, Inc.), 1% glutamine (cat. n^o^ 25030024, Gibco; Thermo Fisher Scientific, Inc.) and 1% penicillin/streptomycin (cat. n° 15,140,122, Gibco; Thermo Fisher Scientific, Inc.) and maintained in a humidified incubator at 37 °C with 5% CO_2_ and 95% (vol/vol) O_2_. SW48 cell line was cultured in Leibovitz’s L-15 medium (cat. n° 11,415–049, Gibco; Thermo Fisher Scientific, Inc.) with 10% fetal bovine serum, 1% glutamine and 1% penicillin/streptomycin and maintained in a humidified incubator at 37 °C with 0% CO_2_ and 100% (vol/vol) O_2_. Cell lines were routinely tested for mycoplasma contamination using the LookOut® Mycoplasma PCR Detection kit (cat. n^o^ MP0035, MilliporeSigma, Merck KGaA).

TRAP1 transient silencing was performed using 80 nM siRNA purchased from Qiagen for 48 h in HCT116 cell line, as previously described [19] and using the same amount of siRNA and Lipofectamine 3000 Transfection Reagent (cat. n^o^ L3000015; Thermo Fisher Scientific, Inc.) for 48 h in SW48 cell line, according to the manufacturer’s instructions. An independent siRNA of TRAP1 (siTRAP1(2)) (cat. n° SI00115164, Qiagen) was used for selected confirmation experiments in HCT116 cells. TFAM transient silencing has been achieved using 80 nM of siRNA purchased from Qiagen (target sequence GGCGGAGTGGCAGGTATATAA, cat. n° SI03105858) for 48 h. Double silencing (TRAP1 and TFAM) was performed pooling 80 nM of each siRNA and the same amount of non-target siRNA (siNEG) was used as a negative control [19].

### RNA extraction and reverse transcription-quantitative PCR (RT-qPCR)

Total RNA isolation, mRNA reverse transcription into cDNA and RT-qPCR set up were performed as previously described [19]. Primers were purchased from Millipore, Sigma or Invitrogen (Thermo Fisher Scientific, Inc.) and sequences for mitochondrial genes are reported in Avolio et al. [20]. α-Tubulin housekeeping gene sequences have been previously listed [19].

### Western blot analysis

Cell pellets were lysed in ice-cold RIPA buffer to isolate whole cell proteins as reported in Landriscina et al. [21], and mitochondrial proteins purification was performed using Qproteome Mitochondria Isolation Kit (cat. n^o^ 37612, Qiagen GmbH) according to the manufacturer’s instructions.

Primary antibodies against TRAP1 (1/1000; cat. n^o^ sc-73604, Santa Cruz Biotechnology, Inc.), ATP8 (1/500; cat. n^o^ ab243667, Abcam), COX2 (Total OXPHOS Human WB Antibody Cocktail 1/1000; cat. n^o^ ab110411, Abcam), ND1 (1/1000; cat. n^o^ GTX34092, GeneTex, Inc.), ND4L (1/1000; cat. n^o^ PA5-103,953, Invitrogen), mtTFA (1/20000; cat. n^o^ ab176558, Abcam), MAPkinase ERK1/2 (1/1000; cat. n°442,704, Calbiochem, Merck KGaA), phospho-p44/42 MAPK (Erk1/2) (Thr202/Tyr204) (E10) (1/1000; cat. n° 9106, Cell Signaling Technology, Inc.), ATP synthase subunit beta (1/500; cat. n^o^ A21351, Invitrogen), GAPDH (1/1000; cat. n^o^ sc-47724, Santa Cruz Biotechnology) and β-actin (1/2000; cat. n^o^ sc-47778, Santa Cruz Biotechnology) were used for immunoblot analysis. Protein expression levels were quantified by densitometric analysis, using ImageJ software v1.53e (National Institutes of Health) and normalized according to the expression of housekeeping genes.

### Analysis of public datasets

Tumour/normal differential expression analysis and correlation analysis were performed employing the online tool GEPIA2 (http://gepia2.cancer-pku.cn). GEPIA2 is a web server for analysing the RNA sequencing expression data of tumours and normal samples from TCGA and the GTEx projects, using a standard processing pipeline [22].

The protein expression analysis was carried out interrogating the UALCAN web resource (http://ualcan.path.uab.edu) using data from Clinical Proteomic Tumour Analysis Consortium (CPTAC) and the International Cancer Proteogenome Consortium (ICPC) datasets [23].

### Oxygen consumption rates assay

Oxygen consumption rate (OCR) in TRAP1-silenced and control HCT116 cells was measured by Hansatech Oxygraph in a thermostatically controlled chamber (T = 37 °C). After 48 h of transfection, a suspension of 15 × 10^6^ cells/ml was added into the reaction chamber. After measurement of the stationary resting oxygen consumption rate (OCR_R_, basal), 1 µg/ml oligomycin was added to determine the ATP-linked respiration (OCR_O_), followed by the addition of 10 µM of the uncoupler FCCP (Carbonyl cyanide-4 (trifluoromethoxy) phenylhydrazone) to measure the maximal respiration activity (ORC_U_). The OCR measurements were corrected for the rotenone (2 µM) respiration and normalized for the cell number.

### Immunofluorescence

HCT116 TRAP1-silenced and control cells (2.5 × 10^4^/0.5 ml) were grown on chamber slides 4 well (Nunc™ Lab-Tek™ II Chamber Slide™ System, cat. n^o^ 154526, Thermo Fisher Scientific, Inc.) for 24 h before staining_._ After incubation, cells were treated with 500 nM MitoTracker Red CMXRos (cat. n^o^ M7512, Invitrogen, Thermo Fisher Scientific, Inc.) for 30 min in the dark, washed with 1X PBS, fixed in 4% paraformaldehyde and then permeabilized using 0.2% Triton X-100. For immunofluorescence, cells were incubated in blocking solution (0.4% BSA, 5% FBS in 1X PBS) for 1 h, in anti-TRAP1 antibody (1/100 overnight at 4 °C; cat. n^o^ sc-73604, Santa Cruz Biotechnology, Inc.) and finally in FITC-conjugated secondary antibody (1/300 45 min at room temperature; cat. n^o^ ab6785, Abcam). Slides were mounted using the mounting medium containing DAPI (cat. n^o^ H-1200–10, Vectashield, Vector Laboratories) and the fluorescence signal was detected with the Leica SP8 confocal microscope (Leica Microsystems).

### mtDNA copy number

Genomic DNA was isolated from cells using QIAamp DNA Mini kit (cat. n^o^ 51304, Qiagen GmbH), according to the manufacturer’s instructions. The mtDNA copy number (mtDNA-CN) was assessed by comparing the amount of mitochondrial DNA (mtDNA) versus nuclear DNA (nDNA) by quantitative real-time PCR (qPCR). Sequences of primers for the mitochondrial genome region D-LOOP and nuclear-encoded gene β-ACTIN (nACTB), purchased from Invitrogen (Thermo Fisher Scientific, Inc.), were as follows: D-LOOP (forward primer 5′-TCACCCTATTAACCACTCACGG-3′, reverse primer 5′-ATACTGCGACATAGGGTGCTC-3′) [24] and nACTB (forward primer 5′-TGAGTGGCCCGCTACCTCTT-3′, reverse primer 5′-CGGCAGAAGAGAGAACCAGTGA-3′).

### Statistical analysis

RT-qPCR and qPCR data were analysed using CFX Maestro software version 1.0 (Bio-Rad Laboratories, Inc.) and reported as mean values of two independent experiments (± SEM). A value of *p* < 0.05 was considered to indicate a statistically significant difference.

Densitometric data were analysed by the unpaired *t*-test using the *t*-test calculator of GraphPad (online version). Data are reported as mean values of two independent experiments (± SD). A value of *p* < 0.05 was considered to indicate a statistically significant difference.

The differential expression analysis (boxplot graph) performed using GEPIA2 was set up on |Log_2_FC| cutoff = 1 and *p*-value cutoff = 0.01 and applying Pearson’s correlation. A value of *p* < 0.05 was considered statistically significant. For the protein expression analysis with the UALCAN web resource, a value of *p* < 0.05 was considered to indicate a statistically significant difference.

OCR data are reported as a mean of fold change OCR values of two independent experiments (± SD). Student’s *t*-test was applied and a *p*-value < 0.05 was considered for significant differences.

Confocal microscopy data are reported as mean of three acquisition fields (± SD). Fluorescence intensity was represented by setting siNEG control sample equal to 100% (for both FITC-TRAP1 and MitoTracker) and the relative fluorescence of TRAP1-silenced samples was calculated accordingly. Data were compared by an unpaired *t*-test using the *t*-test calculator of GraphPad (online version). A value of *p* < 0.05 was considered for statistically significant differences.

## Results

### Transcriptional inverse correlation between TRAP1 and protein-coding mitochondrial genes in human CRC cell lines and colon adenocarcinoma tissues

Based on our previous reports supporting an inhibitory role of TRAP1 on mitochondrial respiration, thus favouring glycolysis [15, 17], we thought to further study TRAP1 as a potential regulator of mitochondrial dynamics in CRC.

**Fig. 1:**
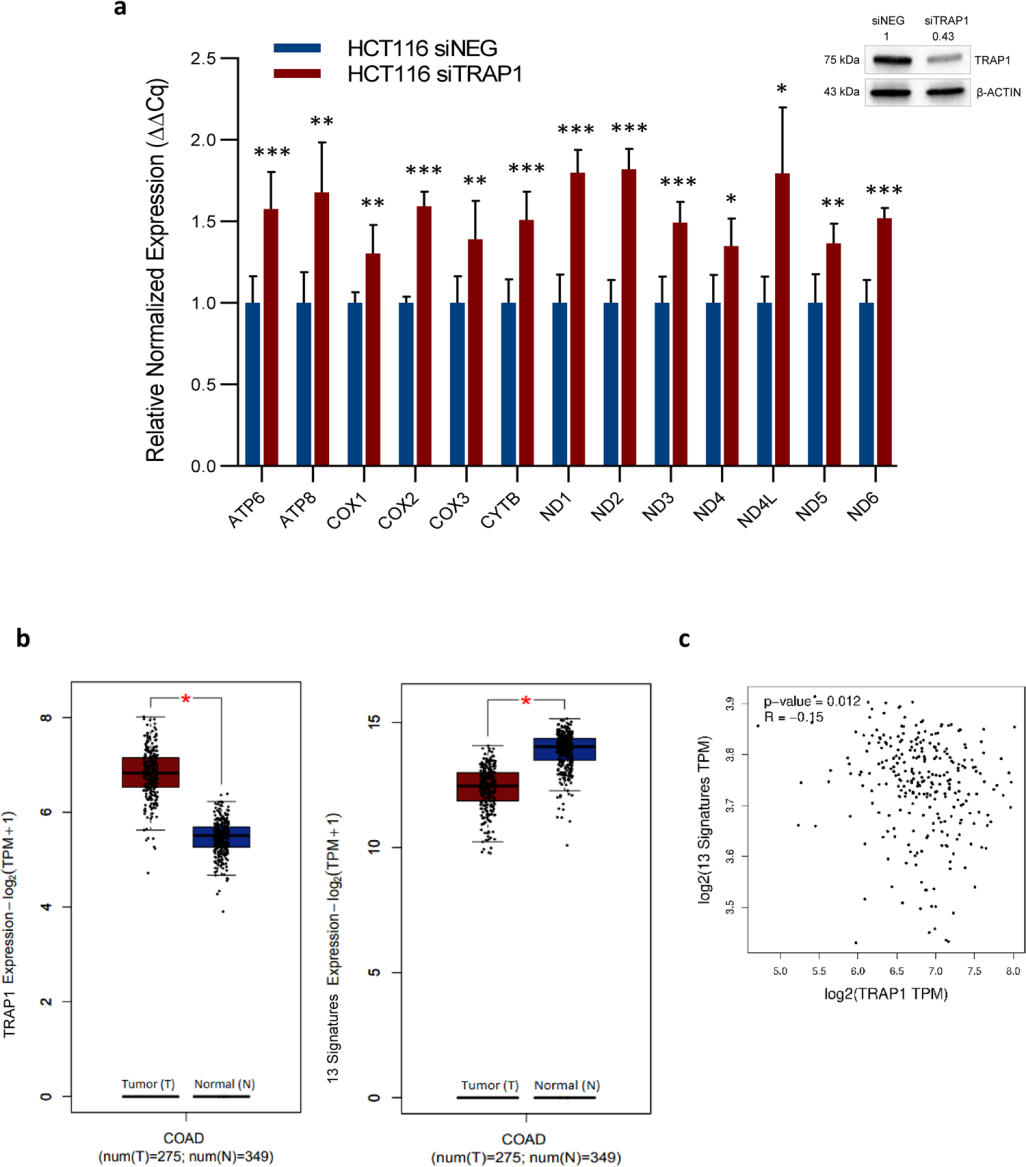
Inverse transcriptional correlation between TRAP1 and protein-coding mt-genes in human CRC HCT116 cells and colon adenocarcinoma tissues. **a** RT-qPCR analysis of protein-coding 13 mt-genes in HCT116 cells transiently transfected with TRAP1 (siTRAP1) or negative control (siNEG) siRNAs. The relative normalized expression is the mean of two independent experiments (± SEM) and *p*-values indicate statistically significant differences (*p < 0.05, **p < 0.01, ***p < 0.001). Insert: western blot and densitometric analysis of TRAP1 protein expression in TRAP1-silenced and control HCT116 cells. **b** Boxplot graphs of TRAP1 (left graph) and protein-coding 13 mt-genes signature (right graph) mRNA expression comparing human normal colorectal mucosa (*n* = 349) and colorectal cancer (*n* = 275) using multiple dataset COAD matched with TCGA normal and GTEx data in GEPIA2 tool (|Log_2_FC| cutoff = 1; *p*-value cutoff = 0.01). **c** Correlation analysis graph between TRAP1 and protein-coding 13 mt-genes transcript levels using TCGA COAD tumour dataset of GEPIA2. Pearson’s linear correlation was applied and *p*-value indicates statistically significant differences (*p* < 0.05)

Thus, HCT116 cells transiently silenced for TRAP1 were used to study the expression of the 13 mitochondrial (mt-) genes coding for polypeptides of the respiratory chain complexes by RT-qPCR. Noteworthy, as previously reported [25], TRAP1 silencing did not increase rates of apoptotic cell death (data not shown). Furthermore, TRAP1 silencing significantly correlated with enhanced expression of all mt-genes tested (*p*-value < 0.05, Fig. 1a) compared to siNEG control. The efficacy of TRAP1 silencing was assessed by parallel western blot analysis (Fig. 1a insert). Additional experiments performed in HCT116 cells using an independent siRNA for TRAP1 (named siTRAP1(2)) confirmed this observation (suppl. Fig. 1a). Also, CRC SW48 cells were identified as a second cell line for parallel experiments. Indeed, TRAP1-silencing (siTRAP1(1)) induced a significant increase in all 13 mt-genes expression compared to siNEG (*p*-value < 0.05, supplementary Fig. 1b) in SW48 cells as observed in HCT116 cells. The transiently transfection efficacy was verified by western blot (suppl. Fig. 1a and b, inserts).

In a translational perspective, GEPIA2 tool was interrogated to analyse, at transcriptional level, TRAP1 expression in human CRC samples (*n* = 275) compared to human normal colorectal mucosa (*n* = 349) and the correlation between TRAP1 and 13 mt-genes expression. As expected, a significant upregulation of TRAP1 expression was observed in cancer tissues (|Log_2_FC| cutoff = 1; *p*-value cutoff = 0.01) (Fig. 1b, left panel), confirming our previous observation obtained using TNMplot database [19]. Conversely, the expression of 13 mt-genes (mt-signature) was significantly downregulated in malignant tissues with respect to normal samples (Fig. 1b, right panel). Also, a correlation analysis between TRAP1 and mt-signature transcript levels was performed using the TCGA COAD tumour dataset of GEPIA2. Applying the Pearson’s linear correlation test, a weak but statistically significant negative correlation was observed (*p*-value < 0.05, Fig. 1c), supporting our finding in human CRC cell lines.

### TRAP1 inversely correlates with mitochondria-encoded proteins levels in human CRC cell lines and tumours

**Fig. 2:**
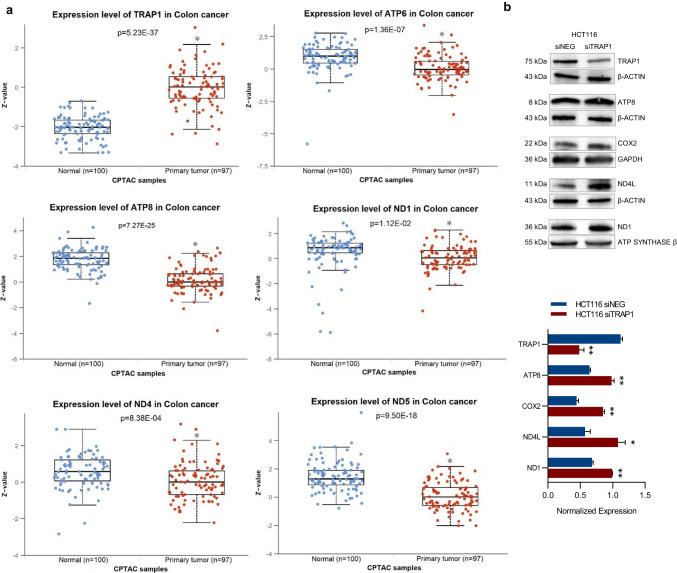
TRAP1 conversely correlates with mitochondria-encoded proteins in human CRC primary tumours and HCT116 cells. **a** Jitter plots of TRAP1 and 5 mt-genes protein expression comparing colorectal primary tumours (*n* = 97) and normal colorectal mucosa (*n* = 100) using CPTAC dataset of UALCAN web resource. *p*-value indicates statistically significant differences (*p < 0.05). **b** Western blot analysis of TRAP1 and mitochondrial proteins ATP8, COX2, ND1 and ND4L in HCT116 cells silenced or not for TRAP1 (upper panel). Densitometric analysis reports the mean of two independent experiments (± SD) and *p*-value indicates statistically significant differences (lower panel) (*p < 0.05, **p < 0.01, ***p < 0.001). GAPDH, β-ACTIN and ATP synthase β were used as housekeeping genes for protein expression normalization

To functionally validate the data shown above, a protein expression analysis was performed interrogating the UALCAN database. Consistently with previously reported GEPIA2 data (this study) and other previous observations by our group [16, 19], TRAP1 was significantly overexpressed in primary CRC (*n* = 97) vs normal samples (*n* = 100) (*p*-value < 0.05, Fig. 2a). Conversely, considering mitochondria-encoded proteins for which proteomic data were available (i.e. ATP6, ATP8, ND1, ND4 and ND5), their protein expression levels were significantly lower in CRC tumour tissues compared with the normal one (*p*-value < 0.05). TRAP1 and mitochondrial-encoded protein levels were also evaluated by stratifying patients according to stage of disease (stages 1–4) and comparing them with normal samples (suppl. Fig. 2). TRAP1 levels were overexpressed independently from stages of disease (*p* < 0.05) and this is consistent with our previous observation that TRAP1 upregulation is an early event in colorectal carcinogenesis occurring at the transition between low- and high-grade adenomas [16]. Mitochondria-encoded proteins were consistently downregulated starting from early stages of the disease and no significant differences were observed among the different stages.

In our experimental cell models, western blot analysis was performed in high vs. low TRAP1 background in HCT116 cells to evaluate the expression of selected mt-genes, i.e. ATP8, COX2, ND1 and ND4L. Consistently with RT-qPCR data, TRAP1 silencing was associated with higher expression of all tested proteins compared to control cells (*p*-value < 0.05, Fig. 2b). These results were also reproduced in HCT116 cells using the additional siTRAP1(2) and in SW48 cells (suppl. Fig. 3a and b, respectively), confirming data previously observed at the transcriptional level.

As respiratory chain complexes are composed by both nucleus- and mitochondria-encoded proteins, in order to verify whether TRAP1 silencing correlates with the modulation of nucleus-encoded proteins as well, a western blot analysis was performed to evaluate the expression levels of selected proteins encoded by nuclear genes under the same experimental conditions. Data showed significantly increased levels of NADH:ubiquinone oxidoreductase subunit B8 (NDUFB8, complex I) and succinate dehydrogenase β (SDHB, complex II) (suppl. Fig. 3c) in HCT116 cells silenced for TRAP1 compared to controls and this is consistent with our previous study suggesting TRAP1 binding to SDH and inhibition of its activity in a context of CRC cells [15]. ATP5A and UQCRC2 protein levels were unchanged in the same experimental conditions (suppl. Fig. 3c).

To further study the impact of this gene expression remodelling on mitochondria metabolic functions, the OCR was evaluated in TRAP1-silenced and control HCT116 cells by oxygraphy (suppl. Fig. 3d). As previously observed in Seahorse experiments [17], lower TRAP1 levels were associated with a significantly higher mitochondria-dependent respiration, and this was more evident under conditions of maximal respiration (OCR_U_) FCCP stimulated. Altogether, these data confirm that TRAP1 silencing shifts CRC cell metabolism toward OXPHOS as previously reported [17] and suggest that this likely occurs also through the modulation of mitochondria-encoded proteins.

### TRAP1 silencing correlates with increased mitochondrial mass and mtDNA-CN in CRC cells

**Fig. 3:**
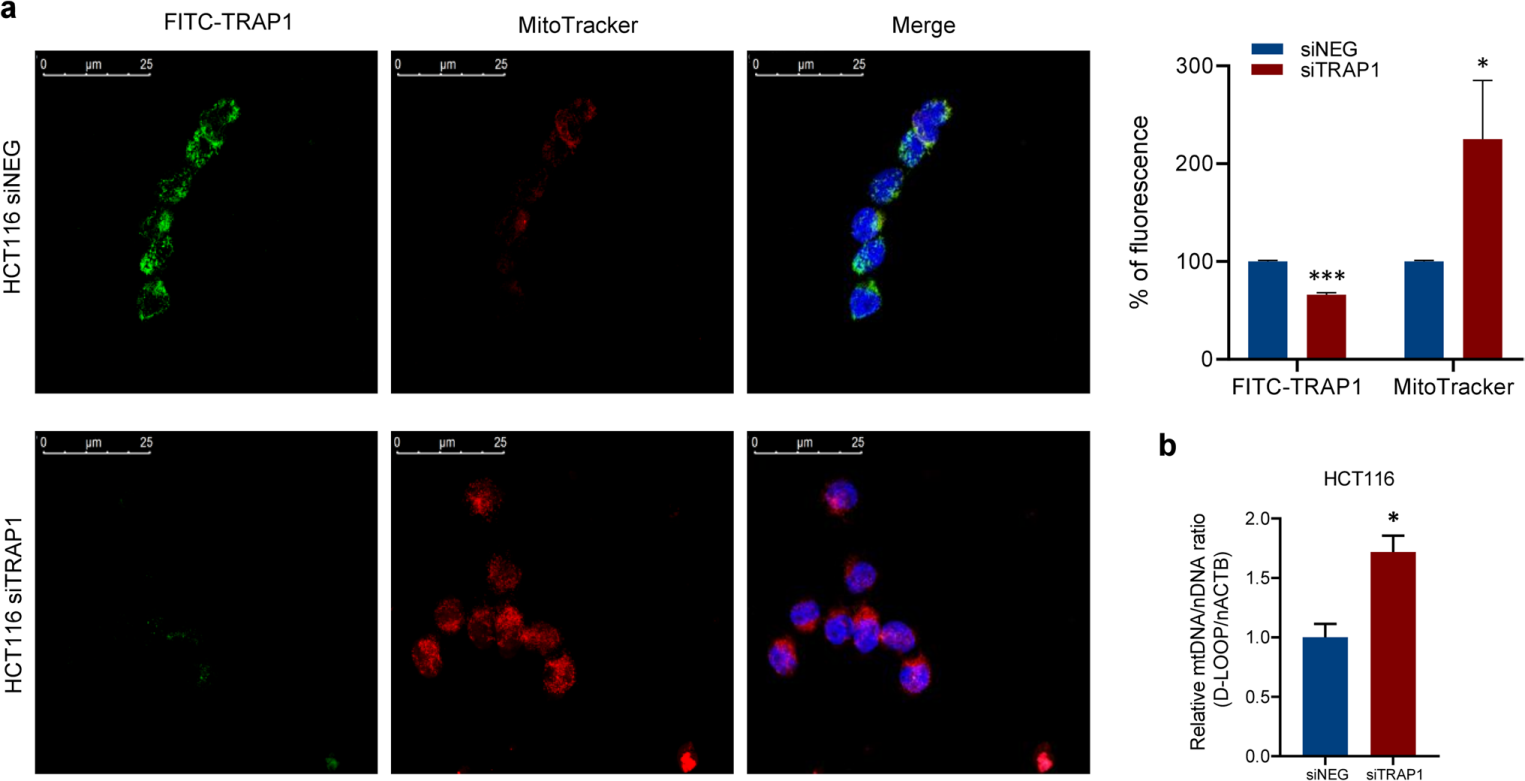
TRAP1 silencing correlates with increased mitochondrial mass and mt-DNA CN in human CRC HCT116 cells. **a** Confocal images of mitochondria from HCT116 cells silenced or not for TRAP1 (left panel): mitochondria stained (red) with MitoTracker, TRAP1 (green) stained with FITC and nuclei (blue) stained with DAPI. Statistical representation of FITC and MitoTracker percentage of fluorescence (mean ± SD) of three acquisition fields (right panel) and *p*-values indicate statistically significant differences (*p < 0.05, **p < 0.01, ***p < 0.001). **b** qPCR analysis of the relative mtDNA/nDNA ratio in HCT116 cells silenced or not for TRAP1. The relative ratio is the mean of two independent experiments (± SEM) and *p*-values indicate statistically significant differences (*p < 0.05, **p < 0.01, ***p < 0.001)

To establish whether the increase in 13 mt-genes and mitochondrial proteins expression was correlated with an increase in mitochondrial content, mitochondria were labelled with MitoTracker in TRAP1-silenced HCT116 cells and analysed by confocal microscopy. Of note, an enhanced MitoTracker signal was observed in TRAP1-silenced cells with respect to siNEG control cells (Fig. 3a left panel), as shown by the statistical analysis reported in the right panel (*p < 0.05, **p < 0.01, ***p < 0.001).

To further study whether TRAP1 may be involved in the regulation of the mitochondrial mass, the mtDNA-CN was assayed by qPCR in our experimental conditions. The relative mtDNA/nDNA ratio was determined measuring the amplification of D-LOOP (mitochondrial genome region) and nACTB, according to the 2-ΔΔCq relative quantification method. The results showed that mtDNA-CN was significantly higher in TRAP1-silenced HCT116 than in control cells (*p*-value < 0.05, Fig. 3b). These data exhibit an increased mitochondrial content in a lower TRAP1 background compared to control, suggesting a role of the chaperone in controlling mitochondrial mass.

### TRAP1 silencing favours PGC1-α/TFAM-mediated mitochondrial biogenesis in CRC cell lines

**Fig. 4:**
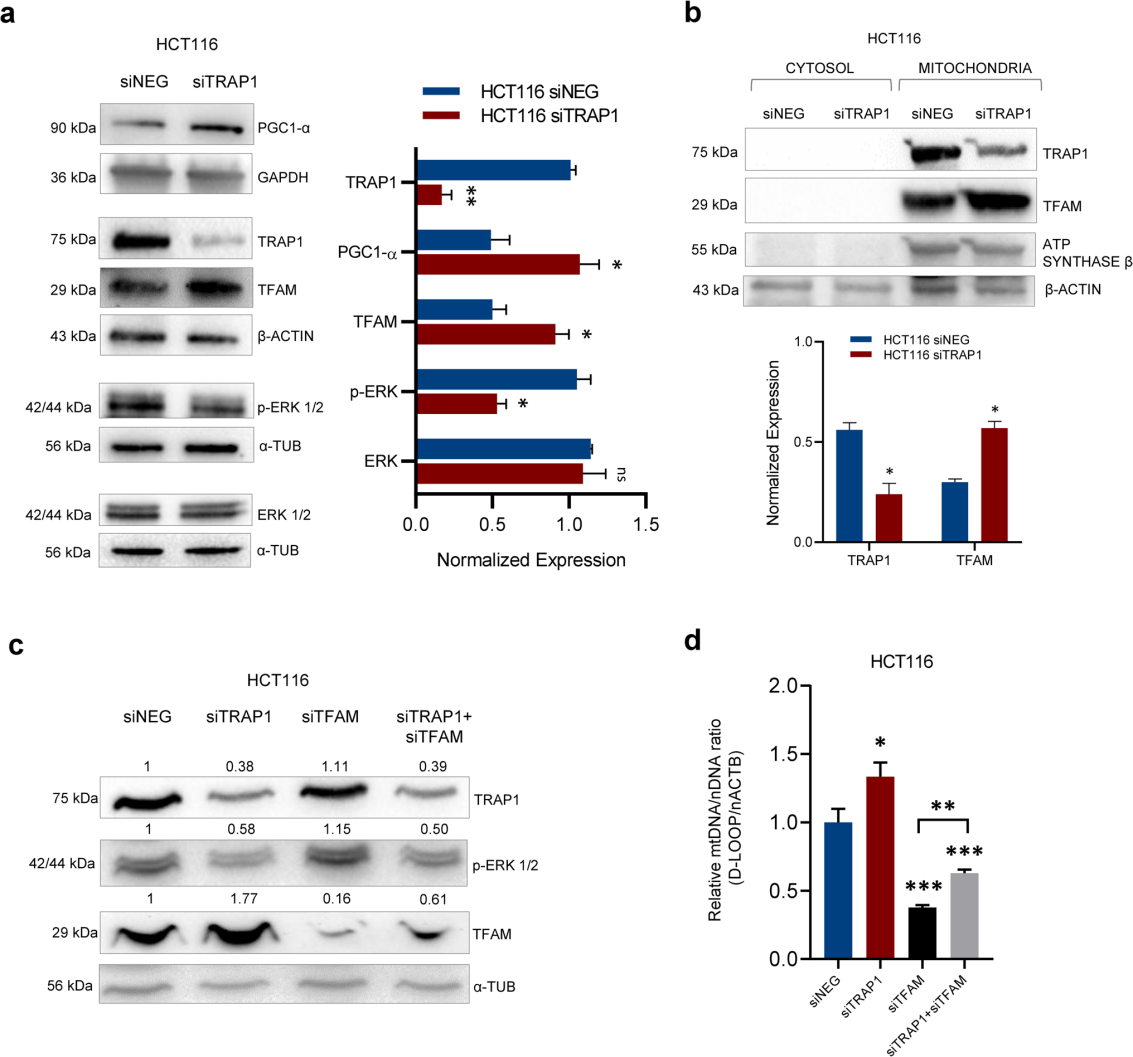
TRAP1 silencing favours MB via PGC1-α/TFAM axis in human CRC HCT116 cells. **a** Western blot analysis of TRAP1, PGC1-α, TFAM, ERK1/2 and phospho-ERK1/2 (p-ERK) proteins in HCT116 cells silenced or not for TRAP1. Densitometric analysis histogram (on the right) reports the mean of two independent experiments (± SD) and *p*-value indicates statistically significant differences (*p < 0.05, **p < 0.01, ***p < 0.001). GAPDH, β-ACTIN and α-TUBULIN were used as housekeeping genes for protein expression normalization. **b** Western blot of TRAP1 and TFAM expression in both mitochondrial and cytosolic compartments in HCT116 cells silenced or not for TRAP1. Densitometric analysis histogram (below) reports the mean of two independent experiments (± SD) and *p*-value indicates statistically significant differences (*p < 0.05, **p < 0.01, ***p < 0.001). ATP synthase β was used as mitochondrial housekeeping genes for protein expression normalization and β-ACTIN as a control of the cytosolic fraction. **c** Western blot analysis of TFAM, TRAP1 and p-ERK1/2 proteins in HCT116 cells silenced for TRAP1 and/or TFAM. Densitometric analysis reports the mean of two independent experiments (± SD) and *p*-value indicates statistically significant differences (*p < 0.05, **p < 0.01, ***p < 0.001). α-TUBULIN was used as housekeeping genes for protein expression normalization. **d** qPCR analysis of the relative mtDNA/nDNA ratio in HCT116 cells silenced for TRAP1 and/or TFAM. The relative ratio is the mean of two independent experiments (± SEM) and *p*-values indicate statistically significant differences (*p < 0.05, **p < 0.01, ***p < 0.001)

Since mitochondrial biogenesis (MB) is the process by which cells increase mitochondrial mass [26] and both mtDNA/nDNA ratio and mt-genes expression levels are considered markers of this process [27, 28], a role of TRAP1 in regulating MB was further investigated in CRC cell lines. Western blot analysis was performed to evaluate the expression changes of two main factors involved in this finely regulated process, i.e. the peroxisome proliferator-activated receptor-γ-coactivator (PGC)1-α and the transcription factor A, mitochondrial (TFAM). Effectively, TRAP1 silencing was associated with higher expression of PGC1-α and TFAM compared to negative control in both HCT116 (*p*-value < 0.05, Fig. 4a) and SW48 (*p*-value < 0.05, suppl. Fig. 4a) cell lines. Considering that TFAM is a nuclear-encoded protein imported into mitochondria, its expression was also evaluated in cytosolic and mitochondrial fractions purified from TRAP1-silenced HCT116 cells. Interestingly, TRAP1 and TFAM proteins were found in the mitochondrial compartment, and TFAM levels significantly increased in TRAP1 silencing conditions (*p*-value < 0.05, Fig. 4b). These data suggest the involvement of TRAP1 in the regulation of MB process through PGC1-α/TFAM axis.

In order to suggest a possible molecular mechanism underlying the regulation of MB associated with lower TRAP1 levels, the involvement of the extracellular signal-regulated kinases 1/2 (ERK1/2) pathway was considered in this process. Indeed, activated ERK1/2 has been shown to reduce basal respiration in mitochondria, ATP production and MB targets [29, 30]. Also, in our previous study, we observed the downregulation of ERK1/2 phosphorylation upon transient TRAP1 silencing in HCT116 [31]. Consistently, western blot analysis showed a significant decrease of phosphorylated ERK1/2 levels in TRAP1-interfered HCT116 and SW48 cell lines compared to control cells (Fig. 4a and suppl. Fig. 4a, respectively), in presence of almost unchanged levels of the non-phosphorylated total protein. The reduced phosphorylation/activation of ERK1/2 in the presence of lower TRAP1 levels also occurred in parallel with the increase of PGC1-α expression and this is consistent with published studies which described an inverse correlation between ERK1/2 phosphorylation status and PGC1-α levels in mouse fibroblast [32] and in physiological and pathological conditions as kidney injury [29].

To prove that the increased mitochondrial content following TRAP1 interference occurs through TFAM regulation, mtDNA-CN was assayed upon TFAM transient silencing in high versus low TRAP1 levels in HCT116 and SW48 cells. Western blot and densitometric analysis showed first the effectiveness of siRNAs alone and in combination compared to negative control in both HCT116 (Fig. 4c) and SW48 (suppl. Fig. 4b) cell lines. Of note, in conditions of simultaneous TRAP1/TFAM silencing, lower TRAP1 levels played a compensatory effect increasing TFAM expression in HCT116 and SW48 cells compared to TFAM-silenced control (siTFAM), which was translated in significant mtDNA-CN variations. Indeed, qPCR analysis of mtDNA-CN revealed a significant inhibition of the relative mtDNA/nDNA ratio following TFAM silencing compared to negative control (*p*-value < 0.05, Fig. 4d and suppl. Fig. 4c), as expected. Instead, the concomitant TRAP1/TFAM silencing partially rescued mtDNA-CN compared to TFAM single siRNA (*p*-value < 0.05, Fig. 4d and suppl. Fig. 4c), consistently with TFAM levels. Interestingly, ERK1/2 phosphorylation status was influenced only by TRAP1 expression levels, suggesting that ERK1/2 phosphorylation is modulated by TRAP1 protein network upstream to TFAM pathway. These data suggest a functional involvement of TRAP1 in regulating mitochondrial content via TFAM signalling pathway.

## Discussion

Mitochondria are the cell powerhouse, responsible for the production of ATP via the tricarboxylic acid cycle (TCA) and OXPHOS, and this drives many cellular processes crucial for the maintenance of metabolic homeostasis [33]. Human mitochondria have their double-stranded circular genome of 16.6 kb in size coding for 37 genes, and among them, 13 encode for proteins of respiratory chain complexes [34]. Unlike nDNA, numerous copies of mtDNA are present in a single mitochondrion and the total number of mitochondria in cells depends on cell type and is finely regulated by energy demand. Contrary to initial Warburg’s hypothesis, mitochondria are not obsolete or useless for cancer cells but maintain their functions [18], ensuring metabolic and cellular plasticity. Indeed, cell metabolism differs in cancer from normal cells, with prominent activation of aerobic glycolysis, reactive oxygen species (ROS) accumulation and anti-apoptotic signalling pathways [35]. In such a context, mitochondria play a crucial role in cancer cell bioenergetics, supporting tumorigenesis from malignant transformation to metastasis formation, with mitochondrial stress response conferring a rapid adaptation to hostile microenvironment conditions [10, 11]. Finally, dysregulation of mitochondrial biogenesis, dynamics, and mitophagy is also crucial in several pathophysiological conditions, including cancer, being responsible for regulation of cell death and apoptosis and being considered a key regulator of oncogenesis [36].

In this complex scenario of molecular processes underlying pro-neoplastic mitochondrial adaptations, TRAP1 has been identified as one of the main regulators of mitochondrial bioenergetics of cancer cells [35]. TRAP1 downregulates OXPHOS upon inhibition of succinate dehydrogenase and cytochrome c oxidase [15, 37] and concomitantly increases Warburg metabolism through the upregulation of glucose transporter 1 (GLUT1), glucose uptake, phosphofructokinase-1 (PFK1) activity and lactate production [17]. Therefore, human malignancies with high TRAP1 level, including CRC, are characterized by a predominant glycolytic phenotype, whereas other human neoplasms characterized by low TRAP1 expression (i.e. ovarian, cervical and renal carcinomas) exhibit a predominant oxidative metabolism [15, 38, 39]. Thus, cancer cells may modulate TRAP1 expression to adapt to stress conditions, control the apoptotic threshold and rewire their metabolism from aerobic glycolysis to OXPHOS and vice versa [14, 17].

Considering these evidences, the present study aimed at investigating TRAP1 involvement in mitochondrial dynamics in CRC cell lines and human CRCs. Our data showed that TRAP1 downregulation correlates with (i) increased expression of all 13 mt-genes coding for respiratory chain complexes subunits at mRNA and protein levels and (ii) increased mitochondrial mass and mtDNA-CN, respectively assayed by immunofluorescence coupled to confocal microscopy and qPCR analyses. Interestingly, the comparative analysis of TRAP1 and 13 mt-genes expression levels in human CRC samples vs normal colorectal mucosa in public datasets supported these evidences, showing an inverse correlation between TRAP1 and mt-signature in malignant tissues with respect to normal tissues both at transcription and translational level. Also, higher levels of mitochondrial proteins, mitochondrial mass and mtDNA-CN following TRAP1 silencing in HCT116 cells occur together with the increase in mitochondria-dependent respiration.

The increased mitochondrial mass in a low TRAP1 background led us to hypothesize a new role of the chaperone in regulating MB. Indeed, TRAP1 downregulation was associated with both increased levels of the MB master regulators PGC-1α and TFAM, and reduced activation of ERK1/2 signalling. Furthermore, TFAM silencing in a low TRAP1 background partially rescued the low mitochondrial mass observed upon TFAM silencing in a high TRAP1 background, suggesting that TFAM could be a downstream effector of TRAP1 in controlling mitochondrial mass, given that it is widely recognized as a key factor for mtDNA packaging, stability and replication [40, 41].

In such a context, our data may propose ERK1/2 signalling as molecular link between TRAP1 and MB regulation mediated by PGC-1α/TFAM axis in CRC cell lines. Indeed, much evidence suggests that activation of ERK1/2 signalling is responsible for the reduction of basal respiration in mitochondria, ATP production and MB targets [29, 30], and that its downregulation occurs under TRAP1 silencing conditions in CRC cell lines [31]. Interestingly, in our experimental conditions, lower TRAP1 expression correlated with reduced activation of ERK1/2 signalling together with increased PGC1-α/TFAM expression. Thus, according to previous studies showing the inverse correlation between p-ERK1/2 and PGC1-α levels in different pathophysiological conditions [29], it is intriguing to hypothesize that ERK signalling modulation may occur downstream to TRAP1 protein network and upstream to PGC-1α/TFAM signalling, representing a regulatory axis of MB in human CRC cells.

In the perspective of TRAP1 biology, these data would candidate the regulation of MB process as additional process used by the molecular chaperone to control metabolic rewiring and adaptation to unfavourable conditions. Indeed, this molecular chaperone regulates a network of client proteins involved in several adaptive mechanisms ranging from quality control on protein synthesis and protection from ER stress [42] to resistance to oxidative stress [43] and apoptosis [14] and metabolic rewiring [17]. Thus, cancer cells modulate the expression of TRAP1 and its protein network in response to hostile conditions and exploit several adaptive mechanisms, including the modulation of mitochondrial dynamics. Consistently, Chae et al. [44] reported that Hsp90 inhibition following Gamitrinib treatment impacts the expression of mitochondrial transcription factors (i.e. TFB1M and TFB2M) and ribosomal proteins associated with RNA translation (i.e. MRPLs, MTG1 and ERAL1) in glioblastoma cells, suggesting that the Hsp90-directed protein folding in mitochondria controls central metabolic networks in tumour cells [44]. However, it is important to underline that TRAP1 regulation of cancer bioenergetics is a multifaceted and complex process, based on the interaction of several signalling networks and that still deserves to be studied at multiple levels.

In such a view, the evidence of an inverse correlation between TRAP1 levels and MB process reinforces the molecular rationale to consider this chaperone and its protein network as novel potential biomarkers to identify tumours with predominant Warburg metabolism and as novel molecular targets to inhibit cancer cell bioenergetics. In the era of personalized medicine, considering the recent design and development of novel TRAP1 inhibitors [45], this hypothesis is extremely intriguing in the perspective to candidate cancer cell metabolism pathways as targets for anticancer therapy.

## Conclusions

The present study highlights the role of TRAP1 in MB via PGC-1α/TFAM signalling pathway in CRC cell lines. This information may provide a rationale for further studies aimed at investigating TRAP1 involvement in MB as additional process in the regulation of cancer bioenergetics in human CRC.

## Supplementary Information

Below is the link to the electronic supplementary material.

Supplementary file1 (PDF 486 kb)

Supplementary file2 (PDF 281 kb)

Supplementary file3 (PDF 545 kb)

Supplementary file4 (PDF 515 kb)

## Data Availability

All data generated or analysed during this study are included in this published article (and in the supplementary files).

## Abbreviations

CRC: Colorectal cancer
MB: Mitochondrial biogenesis
TRAP1: TNF Receptor-associated Protein 1
OXPHOS: Oxidative phosphorylation
PGC-1α: Peroxisome Proliferation-Activated Receptor Gamma Coactivator α1-Alpha
TFAM: Transcription Factor A Mitochondrial
mtDNA-CN: Mitochondrial DNA copy number;
ERK1/2: Extracellular Signal-Regulated Kinases 1/2
