# Supplementary material for: TRAP1 modulates mitochondrial biogenesis via PGC-1α/TFAM signalling pathway in colorectal cancer cells
Source: J Mol Med (Berl). 2024 Aug 29;102(10):1285–96. doi: 10.1007/s00109-024-02479-9 (PMC11416412; doi:10.1007/s00109-024-02479-9)
Supplement: Supplementary file 1 — Supplementary file1 (PDF 486 kb) [file 109_2024_2479_MOESM1_ESM.pdf]

TRAP1 modulates mitochondrial biogenesis via PGC-1α/TFAM signalling pathway in colorectal cancer cells

Giuseppina Bruno[1], Michele Pietrafesa[2], Fabiana Crispo[2], Annamaria Piscazzi[1], Francesca Maddalena[2], Guido Giordano[1], Vincenza Conteduca[1], Marianna Garofoli[1], Almudena Porras[3,4], Franca Esposito[5] and Matteo Landriscina[1].

[1]Medical Oncology and Biomolecular Therapy Unit, Department of Medical and Surgical Sciences, University of Foggia, 71122 Foggia, Italy; [2]Laboratory of Pre-Clinical and Translational Research, IRCCS, Referral Cancer Center of Basilicata, 85028 Rionero in Vulture, Potenza, Italy; [3]Department of Biochemistry and Molecular Biology, Faculty of Pharmacy, Complutense University of Madrid, 28040 Madrid, Spain; [4]Health Research Institute of the Hospital Clínico San Carlos (IdISSC), 28040, Madrid, Spain; [5]Department of Molecular Medicine and Medical Biotechnology, University of Naples Federico II, 80131 Naples, Italy.

Correspondence to:

Professor Matteo Landriscina, Medical Oncology and Biomolecular Therapy Unit, Department of Medical and Surgical Sciences, University of Foggia, Viale Pinto 1 - 71122 Foggia, Italy.

Email: matteo.landriscina@unifg.it

Dr. Giuseppina Bruno, Medical Oncology and Biomolecular Therapy Unit, Department of Medical and Surgical Sciences, University of Foggia, Viale Pinto 1 - 71122 Foggia, Italy.

Email: giuseppina.bruno@unifg.it

Figure Legend

Suppl. Fig. 1

a) RT-qPCR analysis of 13 mt-genes in HCT116 cells transiently transfected with TRAP1(2) (siTRAP1(2)) or negative control (siNEG) siRNAs. p-values indicate statistically significant differences (*p< 0.05, **p< 0.01, ***p< 0.001). Insert: western blot and densitometric analysis of TRAP1 protein expression in TRAP1-silenced HCT116 cells. **b)** RT-qPCR analysis of 13 mt-genes in SW48 cells transiently transfected with TRAP (siTRAP1) or negative control (siNEG) siRNAs. p-values indicate statistically significant differences (*p< 0.05, **p< 0.01, ***p< 0.001). Insert: western blot and densitometric analysis of TRAP1 protein expression in TRAP1-silenced SW48

a

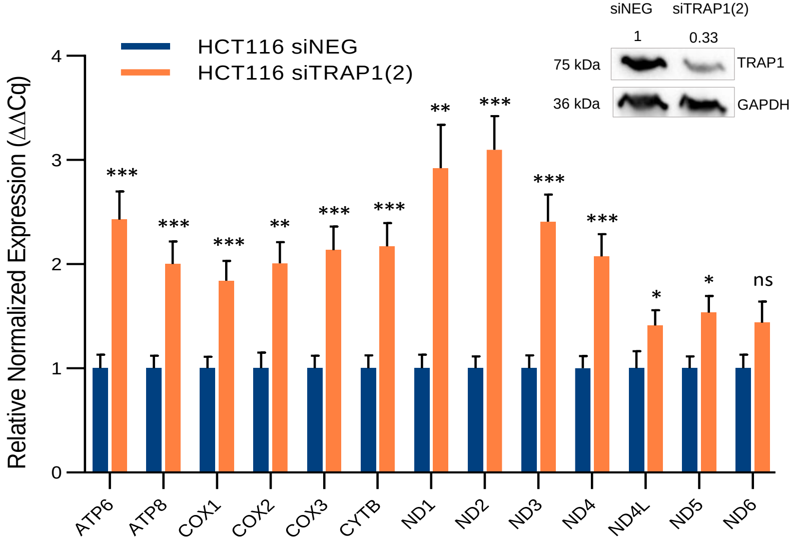
HCT116 siNEG
HCT116 siTRAP1(2)
Relative Normalized Expression (ΔΔCq)
0
1
2
3
4
siNEG
siTRAP1(2)
1
0.33
75 kDa
TRAP1
36 kDa
GAPDH
***
***
***
**
***
***
**
***
***
***
*
*
ns
ATP6
ATP8
COX1
COX2
COX3
CYTB
ND1
ND2
ND3
ND4
ND4L
ND5
ND6

b

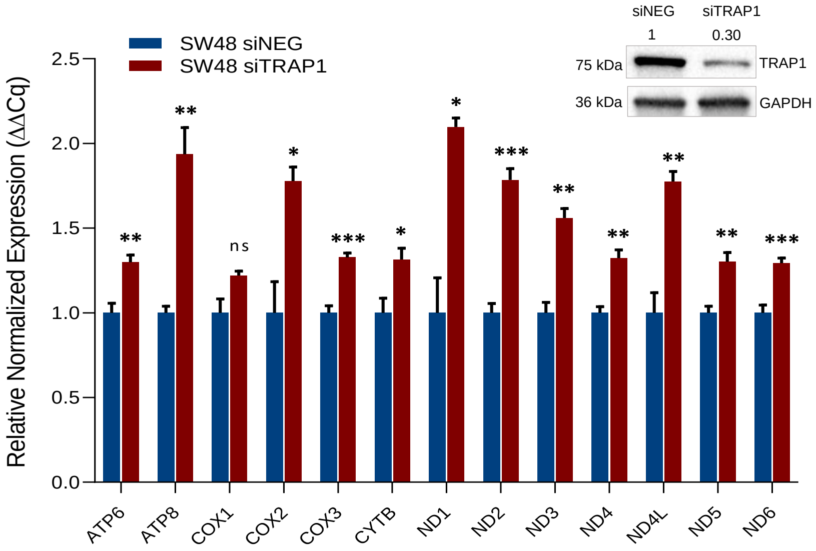
SW48 siNEG
SW48 siTRAP1
Relative Normalized Expression (ΔΔCq)
0.0
0.5
1.0
1.5
2.0
2.5
siNEG
siTRAP1
1
0.30
75 kDa
TRAP1
36 kDa
GAPDH
**
**
ns
*
***
*
*
***
**
**
**
**
***
ATP6
ATP8
COX1
COX2
COX3
CYTB
ND1
ND2
ND3
ND4
ND4L
ND5
ND6
