# Supplementary material for: TRAP1 modulates mitochondrial biogenesis via PGC-1α/TFAM signalling pathway in colorectal cancer cells
Source: J Mol Med (Berl). 2024 Aug 29;102(10):1285–96. doi: 10.1007/s00109-024-02479-9 (PMC11416412; doi:10.1007/s00109-024-02479-9)
Supplement: Supplementary file 2 — Supplementary file2 (PDF 281 kb) [file 109_2024_2479_MOESM2_ESM.pdf]

TRAP1 modulates mitochondrial biogenesis via PGC-1α/TFAM signalling pathway in colorectal cancer cells

Giuseppina Bruno[1], Michele Pietrafesa[2], Fabiana Crispo[2], Annamaria Piscazzi[1], Francesca Maddalena[2], Guido Giordano[1], Vincenza Conteduca[1], Marianna Garofoli[1], Almudena Porras[3,4], Franca Esposito[5] and Matteo Landriscina[1].

[1]Medical Oncology and Biomolecular Therapy Unit, Department of Medical and Surgical Sciences, University of Foggia, 71122 Foggia, Italy;

[2]Laboratory of Pre-Clinical and Translational Research, IRCCS, Referral Cancer Center of Basilicata, 85028 Rionero in Vulture, Potenza, Italy;

[3]Department of Biochemistry and Molecular Biology, Faculty of Pharmacy, Complutense University of Madrid, 28040 Madrid, Spain;

[4]Health Research Institute of the Hospital Clínico San Carlos (IdISSC), 28040, Madrid, Spain;

[5]Department of Molecular Medicine and Medical Biotechnology, University of Naples Federico II, 80131 Naples, Italy.

Correspondence to:

Professor Matteo Landriscina, Medical Oncology and Biomolecular Therapy Unit, Department of Medical and Surgical Sciences, University of Foggia, Viale Pinto 1 - 71122 Foggia, Italy.

Email: matteo.landriscina@unifg.it

Dr. Giuseppina Bruno, Medical Oncology and Biomolecular Therapy Unit, Department of Medical and Surgical Sciences, University of Foggia, Viale Pinto 1 - 71122 Foggia, Italy.

Email: giuseppina.bruno@unifg.it

Figure Legend

Suppl. Fig. 2

a) Jitter plots of TRAP1 and 5 mt-genes protein expression comparing colorectal primary tumours at different stages of disease (stage 1 n=10, stage 2 n=39, stage 3 n=40, stage 4 n=8) and normal colorectal mucosa (n=100) using CPTAC dataset of UALCAN web resource. p-value indicate statistically significant differences (*$p < 0.05$).

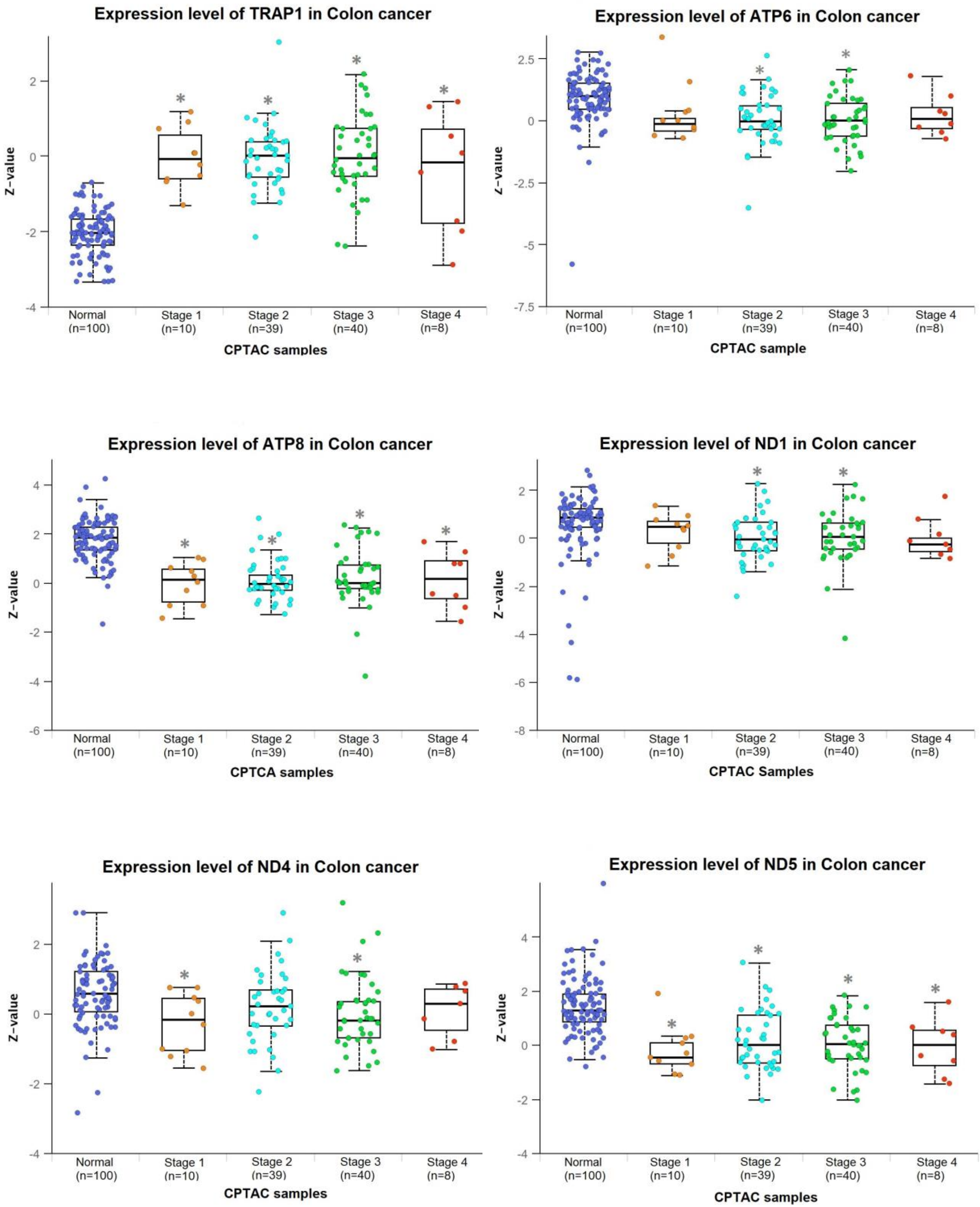
Expression level of TRAP1 in Colon cancer
Z-value
CPTAC samples
Normal (n=100)
Stage 1 (n=10)
Stage 2 (n=39)
Stage 3 (n=40)
Stage 4 (n=8)
Expression level of ATP6 in Colon cancer
Z-value
CPTAC sample
Expression level of ATP8 in Colon cancer
Z-value
CPTCA samples
Expression level of ND1 in Colon cancer
Z-value
CPTAC Samples
Expression level of ND4 in Colon cancer
Z-value
CPTAC samples
Expression level of ND5 in Colon cancer
Z-value
CPTAC samples
