# Supplementary material for: TRAP1 modulates mitochondrial biogenesis via PGC-1α/TFAM signalling pathway in colorectal cancer cells
Source: J Mol Med (Berl). 2024 Aug 29;102(10):1285–96. doi: 10.1007/s00109-024-02479-9 (PMC11416412; doi:10.1007/s00109-024-02479-9)
Supplement: Supplementary file 3 — Supplementary file3 (PDF 545 kb) [file 109_2024_2479_MOESM3_ESM.pdf]

**TRAP1 modulates mitochondrial biogenesis via PGC-1α/TFAM signalling pathway in colorectal cancer cells**

Giuseppina Bruno[1], Michele Pietrafesa[2], Fabiana Crispo[2], Annamaria Piscazzi[1], Francesca Maddalena[2], Guido Giordano[1], Vincenza Conteduca[1], Marianna Garofoli[1], Almudena Porras[3,4], Franca Esposito[5] and Matteo Landriscina[1].

[1]Medical Oncology and Biomolecular Therapy Unit, Department of Medical and Surgical Sciences, University of Foggia, 71122 Foggia, Italy;

[2]Laboratory of Pre-Clinical and Translational Research, IRCCS, Referral Cancer Center of Basilicata, 85028 Rionero in Vulture, Potenza, Italy;

[3]Department of Biochemistry and Molecular Biology, Faculty of Pharmacy, Complutense University of Madrid, 28040 Madrid, Spain;

[4]Health Research Institute of the Hospital Clínico San Carlos (IdISSC), 28040, Madrid, Spain;

[5]Department of Molecular Medicine and Medical Biotechnology, University of Naples Federico II, 80131 Naples, Italy.

**Correspondence to:**

Professor Matteo Landriscina, Medical Oncology and Biomolecular Therapy Unit, Department of Medical and Surgical Sciences, University of Foggia, Viale Pinto 1 - 71122 Foggia, Italy.

Email: matteo.landriscina@unifg.it

Dr. Giuseppina Bruno, Medical Oncology and Biomolecular Therapy Unit, Department of Medical and Surgical Sciences, University of Foggia, Viale Pinto 1 - 71122 Foggia, Italy.

Email: giuseppina.bruno@unifg.it

**Figure Legend**

**Suppl. Fig. 3**

**a)** Western blot analysis of TRAP1,.ATP8, COX2, ND1 and ND4L mitochondrial proteins in HCT116 cells silenced or not for TRAP1 using siTRAP1(2). GAPDH was used as housekeeping gene for protein expression normalization. Densitometric analysis results are reported and p-values indicate statistically significant differences (*$p < 0.05$, **$p < 0.01$, ***$p < 0.001$). Protein lysates are the same shown in suppl. fig. 1a insert. **b)** Western blot analysis of TRAP1, ATP8, COX2, ND1 and ND4L mitochondrial proteins in SW48 cells silenced or not for TRAP1. GAPDH and α-TUBULIN were used as housekeeping genes for protein expression normalization. Densitometric analysis results are reported and p-values indicate statistically significant differences (*$p < 0.05$, **$p < 0.01$, ***$p < 0.001$). Protein lysates are the same shown in suppl. fig. 1b insert. **c)** Western blot analysis of ATP5A, UQCRC2, SDHB, COX2 and NDUFB8 proteins in HCT116 cells silenced or not for TRAP1 incubating Total OXPHOS Human WB Antibody Cocktail. GAPDH was used as housekeeping gene for protein expression normalization. Densitometric analysis (left panel) reports the mean of two independent experiments (±SD) and p-values indicate statistically significant differences (*$p < 0.05$, **$p < 0.01$, ***$p < 0.001$). **d)** Respirometry analysis of HCT116 cells silenced or not for TRAP1. Data are reported as a fold-change of oxygen consumption rate (OCR) of two independent experiments (±SD) and p-values indicate statistically significant differences (*$p < 0.05$, **$p < 0.01$, ***$p < 0.001$).

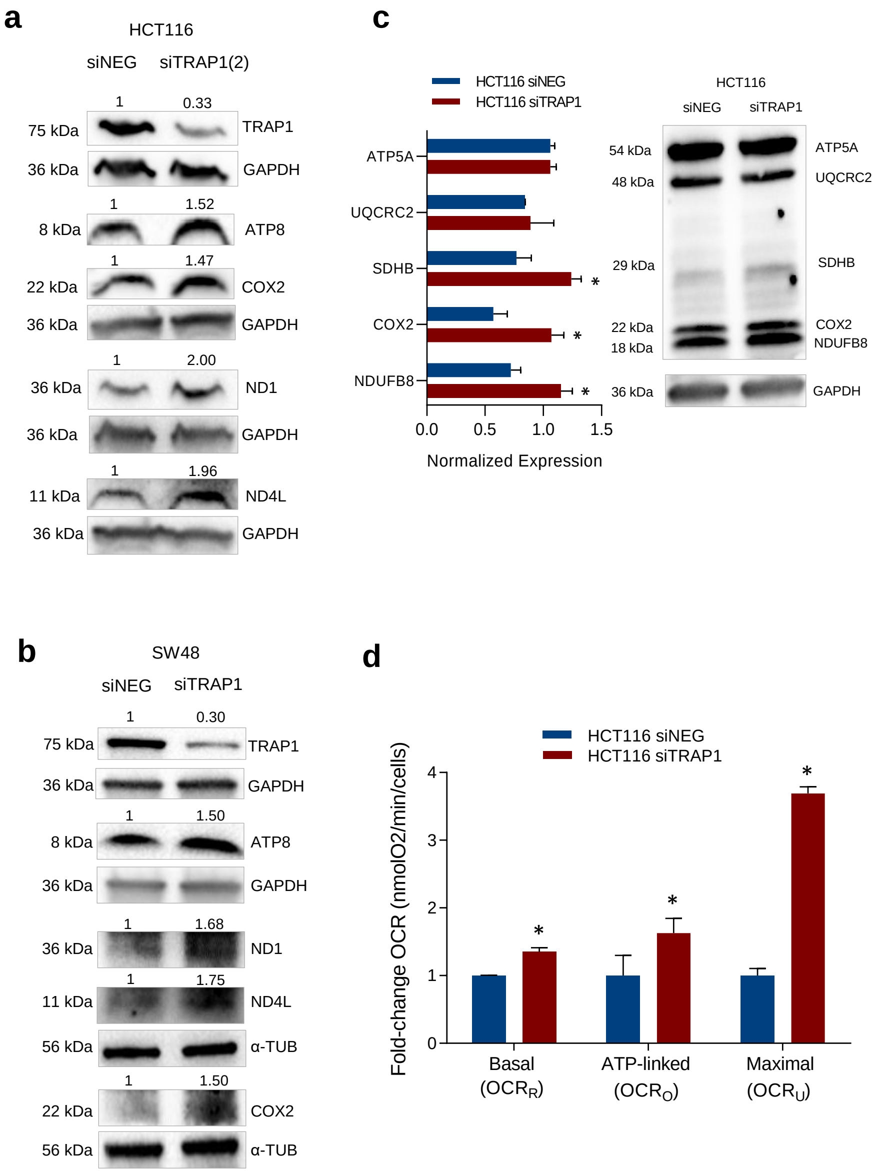
a
HCT116
siNEG
siTRAP1(2)
1
0.33
75 kDa
TRAP1
36 kDa
GAPDH
1
1.52
8 kDa
ATP8
1
1.47
22 kDa
COX2
36 kDa
GAPDH
1
2.00
36 kDa
ND1
36 kDa
GAPDH
1
1.96
11 kDa
ND4L
36 kDa
GAPDH
b
SW48
siNEG
siTRAP1
1
0.30
75 kDa
TRAP1
36 kDa
GAPDH
1
1.50
8 kDa
ATP8
36 kDa
GAPDH
1
1.68
36 kDa
ND1
1
1.75
11 kDa
ND4L
56 kDa
α-TUB
1
1.50
22 kDa
COX2
56 kDa
α-TUB
c
HCT116 siNEG
HCT116 siTRAP1
ATP5A
UQCRC2
SDHB
COX2
NDUFB8
*
*
*
0.0
0.5
1.0
1.5
Normalized Expression
HCT116
siNEG
siTRAP1
54 kDa
ATP5A
48 kDa
UQCRC2
29 kDa
SDHB
22 kDa
COX2
18 kDa
NDUFB8
36 kDa
GAPDH
d
HCT116 siNEG
HCT116 siTRAP1
Fold-change OCR (nmolO2/min/cells)
0
1
2
3
4
*
*
*
Basal
(OCRR)
ATP-linked
(OCRO)
Maximal
(OCRU)
