# Supplementary material for: TRAP1 modulates mitochondrial biogenesis via PGC-1α/TFAM signalling pathway in colorectal cancer cells
Source: J Mol Med (Berl). 2024 Aug 29;102(10):1285–96. doi: 10.1007/s00109-024-02479-9 (PMC11416412; doi:10.1007/s00109-024-02479-9)
Supplement: Supplementary file 4 — Supplementary file4 (PDF 515 kb) [file 109_2024_2479_MOESM4_ESM.pdf]

TRAP1 modulates mitochondrial biogenesis via PGC-1α/TFAM signalling pathway in colorectal cancer cells

Giuseppina Bruno[1], Michele Pietrafesa[2], Fabiana Crispo[2], Annamaria Piscazzi[1], Francesca Maddalena[2], Guido Giordano[1], Vincenza Conteduca[1], Marianna Garofoli[1], Almudena Porras[3,4], Franca Esposito[5] and Matteo Landriscina[1].

[1]Medical Oncology and Biomolecular Therapy Unit, Department of Medical and Surgical Sciences, University of Foggia, 71122 Foggia, Italy; [2]Laboratory of Pre-Clinical and Translational Research, IRCCS, Referral Cancer Center of Basilicata, 85028 Rionero in Vulture, Potenza, Italy; [3]Department of Biochemistry and Molecular Biology, Faculty of Pharmacy, Complutense University of Madrid, 28040 Madrid, Spain; [4]Health Research Institute of the Hospital Clínico San Carlos (IdISSC), 28040, Madrid, Spain; [5]Department of Molecular Medicine and Medical Biotechnology, University of Naples Federico II, 80131 Naples, Italy.

Correspondence to:

Professor Matteo Landriscina, Medical Oncology and Biomolecular Therapy Unit, Department of Medical and Surgical Sciences, University of Foggia, Viale Pinto 1 - 71122 Foggia, Italy.

Email: matteo.landriscina@unifg.it

Dr. Giuseppina Bruno, Medical Oncology and Biomolecular Therapy Unit, Department of Medical and Surgical Sciences, University of Foggia, Viale Pinto 1 - 71122 Foggia, Italy.

Email: giuseppina.bruno@unifg.it

Figure Legend

Suppl. Fig. 4

a) Western blot analysis of PGC1-α, TFAM, ERK1/2 and p-ERK1/2 in SW48 cells silenced or not for TRAP1. GAPDH and α-TUBULIN were used as housekeeping genes for protein expression normalization. Densitometric analysis results are reported. **b)** Western blot analysis of TRAP1, TFAM and p-ERK1/2 proteins in SW48 cells silenced for TRAP1 and/or TFAM. α-TUBULIN was used as housekeeping genes for protein expression normalization. Densitometric analysis results are reported. **e)** qPCR analysis of the relative mtDNA/nDNA ratio in SW48 cells silenced for TRAP1 and/or TFAM. p-values indicate statistically significant differences (*p< 0.05, **p< 0.01, ***p< 0.001).

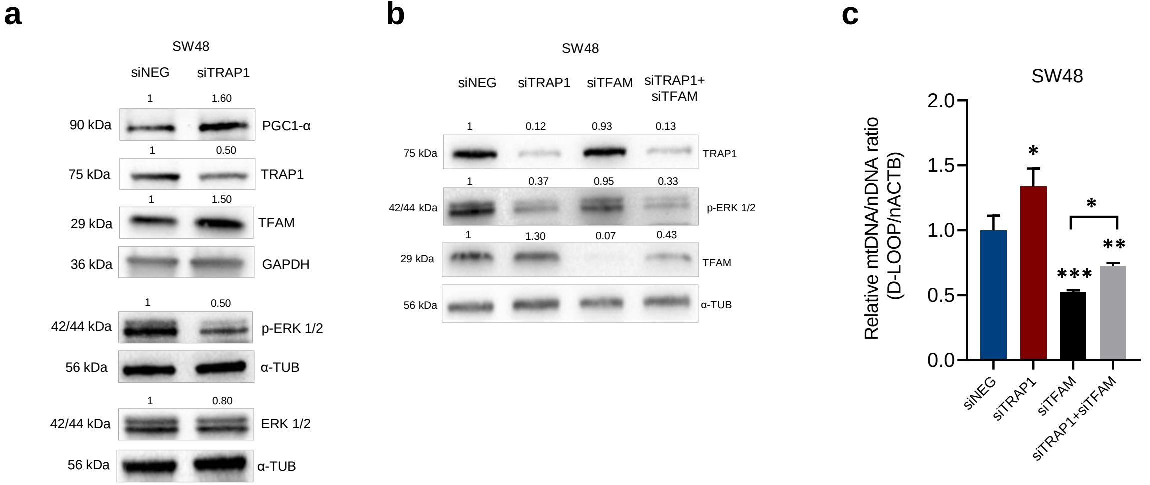
a
SW48
siNEG siTRAP1
1 1.60
90 kDa PGC1-α
1 0.50
75 kDa TRAP1
1 1.50
29 kDa TFAM
36 kDa GAPDH
1 0.50
42/44 kDa p-ERK 1/2
56 kDa α-TUB
1 0.80
42/44 kDa ERK 1/2
56 kDa α-TUB
b
SW48
siNEG siTRAP1 siTFAM siTRAP1+ siTFAM
1 0.12 0.93 0.13
75 kDa TRAP1
1 0.37 0.95 0.33
42/44 kDa p-ERK 1/2
1 1.30 0.07 0.43
29 kDa TFAM
56 kDa α-TUB
c
SW48
Relative mtDNA/nDNA ratio (D-LOOP/nACTB)
2.0
1.5
1.0
0.5
0.0
*
*
***
**
siNEG
siTRAP1
siTFAM
siTRAP1+siTFAM
